# Innate immune sensor LGP2 is cleaved by the Leader protease of foot-and-mouth disease virus

**DOI:** 10.1371/journal.ppat.1007135

**Published:** 2018-06-29

**Authors:** Miguel Rodríguez Pulido, María Teresa Sánchez-Aparicio, Encarnación Martínez-Salas, Adolfo García-Sastre, Francisco Sobrino, Margarita Sáiz

**Affiliations:** 1 Centro de Biología Molecular Severo Ochoa, CSIC-UAM, Madrid, Spain; 2 Department of Microbiology, Icahn School of Medicine at Mount Sinai, New York, United States of America; 3 Global Health and Emerging Pathogens Institute, Icahn School of Medicine at Mount Sinai, New York, United States of America; 4 Department of Medicine, Division of Infectious Diseases, Icahn School of Medicine at Mount Sinai, New York, United States of America; University of California, Irvine, UNITED STATES

## Abstract

The RNA helicase LGP2 (Laboratory of Genetics and Physiology 2) is a non-signaling member of the retinoic acid-inducible gene-I (RIG-I)-like receptors (RLRs), whose pivotal role on innate immune responses against RNA viruses is being increasingly uncovered. LGP2 is known to work in synergy with melanoma differentiation-associated gene 5 (MDA5) to promote the antiviral response induced by picornavirus infection. Here, we describe the activity of the foot-and-mouth disease virus (FMDV) Leader protease (Lpro) targeting LGP2 for cleavage. When LGP2 and Lpro were co-expressed, cleavage products were observed in an Lpro dose-dependent manner while co-expression with a catalytically inactive Lpro mutant had no effect on LGP2 levels or pattern. We further show that Lpro localizes and immunoprecipitates with LGP2 in transfected cells supporting their interaction within the cytoplasm. Evidence of LGP2 proteolysis was also detected during FMDV infection. Moreover, the inhibitory effect of LGP2 overexpression on FMDV growth observed was reverted when Lpro was co-expressed, concomitant with lower levels of IFN-β mRNA and antiviral activity in those cells. The Lpro target site in LGP2 was identified as an RGRAR sequence in a conserved helicase motif whose replacement to EGEAE abrogated LGP2 cleavage by Lpro. Taken together, these data suggest that LGP2 cleavage by the Leader protease of aphthoviruses may represent a novel antagonistic mechanism for immune evasion.

## Author summary

Foot-and-mouth disease virus (FMDV) is the causative agent of a devastating disease affecting livestock worldwide. FMDV is considered an extremely successful pathogen able to replicate and spread rapidly among its hosts. The induction of type-I interferon (IFN) response is a crucial event in mammalian cells against infections, and viruses have evolved a variety of mechanisms to avoid it. LGP2 (Laboratory of Genetics and Physiology 2) is a cellular protein involved in sensing viral RNA during infection and plays a relevant role on regulation of the signaling pathways leading to IFN induction. Here we show that LGP2 is specifically targeted for cleavage by the FMDV-encoded Leader protease (Lpro) and a correlation with a decrease in the IFN levels induced in infected cells. Our data unveil a new viral mechanism of immune evasion based on direct cleavage of LGP2 by the Leader proteases of aphthoviruses. To our knowledge this is the first report of the proteolytic cleavage of LGP2 by a virally-encoded protease. Our findings will contribute to a better understanding of the virus-host interplay involved in pathogenesis and to the development of more efficient strategies to combat infectious diseases.

## Introduction

Antiviral response against RNA viruses greatly relies on detection of infection by cytoplasmic sensors. Among the different pattern-recognition receptors (PRRs) involved in antiviral immunity, the retinoic acid-inducible gene-I (RIG-I)-like receptors (RLRs), recognize non-self RNA species derived from viral infection triggering the downstream signaling cascade leading to type-I interferon (IFN) secretion and host antiviral response [1,2]. RLRs are ubiquitous cytosolic RNA helicases including RIG-I, melanoma differentiation-associated gene 5 (MDA5) and LGP2 (Laboratory of Genetics and Physiology 2). All three RLRs share a DExD/H box RNA helicase domain and a C-terminal domain (CTD). The helicase domain and the CTD bind to viral RNA, CTD being essential for the specific recognition of RNA substrate features. The helicase domain generally functions to coordinate RNA binding, ATP hydrolysis, and conformational rearrangements upon RNA recognition [2,3]. The RLRs share the ability to detect molecular signatures of virus infection, but differ in both their RNA recognition specificity and signaling properties. RIG-I senses primarily 5´-triphosphate blunt-end dsRNA, while MDA5 is activated by long dsRNA, consequently responding to different but overlapping sets of viruses [4,5]. LGP2 has the highest RNA binding affinity of the RLRs, and has the ability to recognize diverse dsRNAs, regardless of the presence of 5´-triphosphate or RNA length [6]. RIG-I and MDA5 contain N-terminal tandem caspase activation and recruitment domains (CARDs) which upon recognition of viral RNA, interact with the CARD of the mitochondrial activator of virus signaling (MAVS) protein, the essential adaptor molecule for RLR signaling. LGP2 lacks the N-terminal CARDs and then independent signaling activity. However, LGP2 is known to be widely involved in viral RNA recognition and regulation during innate immune responses, remaining the most enigmatic member of the RLR family [7]. Both negative and positive regulatory roles have been reported for LGP2 in antiviral immunity. An enhancing effect on MDA5-mediated signaling was found when LGP2 was present at low cellular concentrations. According to a model based on a concentration dependent biphasic switch, at early stages of infection low levels of LGP2 would enhance MDA5-mediated antiviral signaling, but as infection progresses and LGP2 production is induced by IFN, LGP2 would act as a negative feedback regulator inhibiting MDA5 signaling [7–9]. Single molecule RNA binding experiments and biochemical analysis revealed that ATP hydrolysis activity is required to enable LGP2 to efficiently engage diverse dsRNA species, and for enhancement of MDA5 signaling [8]. An RNA- and virus-independent inhibitory role for LGP2 in antiviral signaling has also been reported, likely involving CARD-independent interaction with MAVS by competition with an essential kinase for binding and interfering with downstream signaling [10].

Foot-and-mouth disease virus (FMDV) is the etiologic agent of a highly infectious vesicular disease affecting swine, cattle and other domestic and wild cloven-hoofed animals worldwide [11,12]. FMDV is included in the *Aphthovirus* genus of the *Picornaviridae* family. Picornaviruses are small non-enveloped viruses and their capsids enclose a single-stranded RNA genome coding for a polyprotein which is subsequently cleaved by viral proteases to yield the different viral proteins. MDA5 is involved in recognizing the dsRNA synthesized during picornavirus replication [13] and experimental evidence using lentivirus-driven RNA interference supports that FMDV is sensed by MDA5 in porcine epithelial cells [14].

During co-evolution with their hosts viruses have acquired strategies to actively counteract host antiviral responses [15–17] and the balance between innate response and viral antagonism may determine the outcome of disease and pathogenesis. A repertoire of mechanisms aimed at confronting the host IFN response has been described for FMDV, most of them involving the proteolytic activity of the two virally encoded Leader and 3C proteases [18,19].

The FMDV Leader protease (Lpro) is the first protein encoded in the ORF, a papain-like cysteine protease which is present as two different forms, Lab and Lb, generated by translation initiation at two in-frame AUG codons separated 84 nt on the viral RNA [20] and subsequent intramolecular self-processing. Both forms of Lpro are active but Lbpro is more efficiently translated and abundant in infected cells [21]. FMDV Lpro impairs cap-dependent translation through cleavage of initiation factor eIF4G, leading to a translational host shut-off [22,23] and plays an important role in viral pathogenesis. Several cellular proteins have been identified as Lpro targets [24] and Lpro activity is known to disrupt signaling pathways involved in host defenses, like degradation of the p65 subunit of NF-κB and suppression of IFN-β and inflammatory chemokines by reduction of IRF-3/7 expression [25,26]. The deubiquitinase activity of Lpro is also known to cleave ubiquitin moieties from critical signaling proteins of the type-I IFN signaling pathway, such as RIG-I, TBK1, TRAF3, and TRAF6 [27].

A few reports have identified LGP2 as a potential target for viral antagonism. The paramyxovirus V protein binds to the helicase domains of both MDA5 and LGP2 disrupting their enzymatic activity [28]. A recent work describes the interaction between the Nonstructural Protein 3 (NS3) encoded by the hepatitis C virus (HCV) and the helicase domain of LGP2 by quantitative micro-spectroscopic imaging (Q-MSI) [29]. Overexpression of LGP2 has been shown to reduce FMDV growth and interaction of LGP2 with non-structural protein 2B has been detected by immunoprecipitation experiments [30].

Here, we show that FMDV Lpro targets LGP2 helicase for cleavage, resulting in lower levels of IFN-β and antiviral activity in co-transfected cells. No evidence of proteolysis could be detected with a catalytically inactive version of Lpro. The Lpro target sequence in LGP2 was identified as an RGRAR motif which is part of the conserved helicase motif VI of LGP2. Direct interaction between both proteins was evidenced by immunoprecipitation and co-localization assays. LGP2 processing was also detected during FMDV infection, suggesting that LGP2 cleavage by the Leader protease may be a mechanism developed by aphthoviruses to counteract the host immune response. This is the first report of LGP2 proteolytic cleavage exerted by a viral protease and unveils a novel role for the FMDV leader protease on immune evasion.

## Results

### LGP2 is a target for the FMDV Leader protease

**Fig 1:**
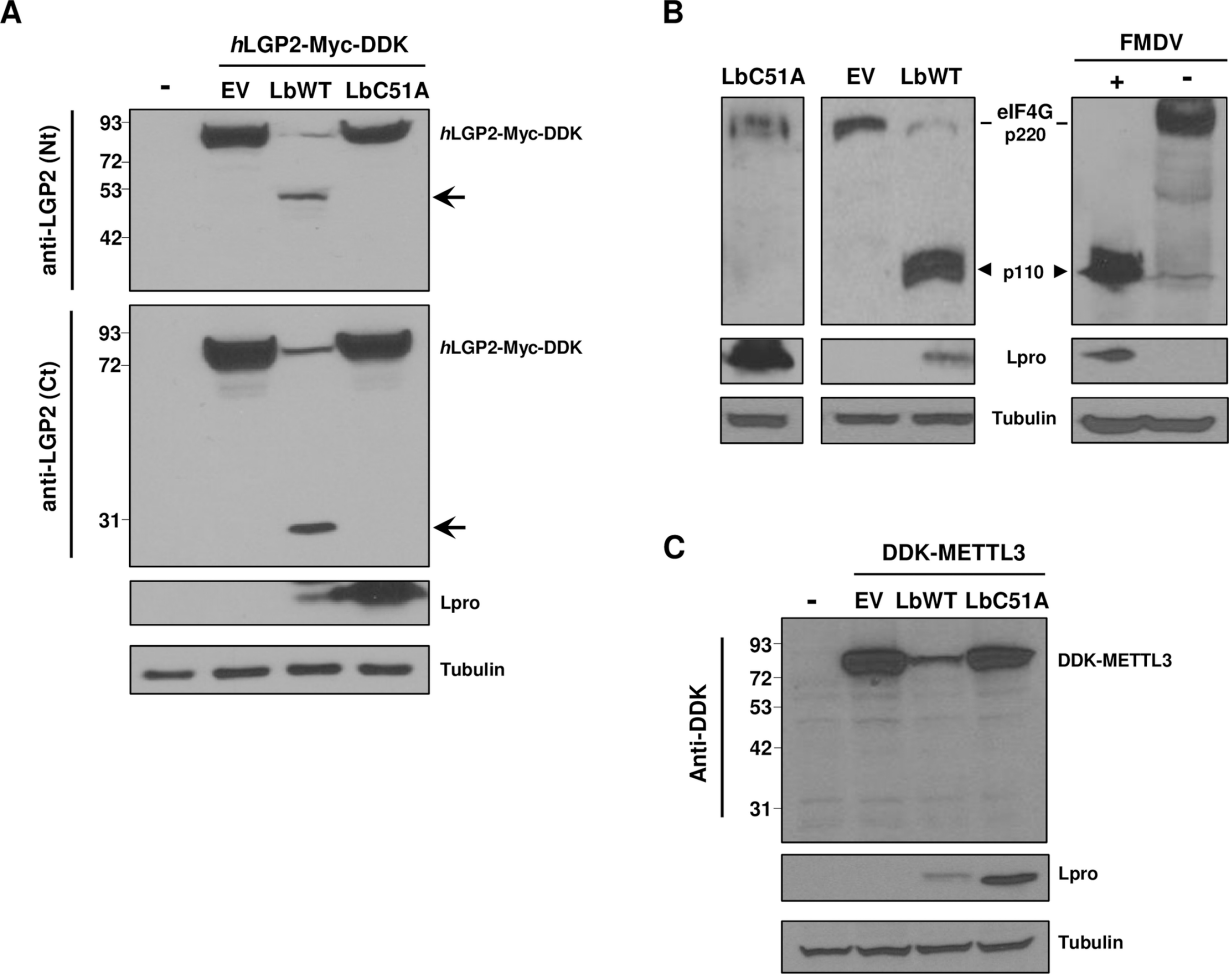
Effect of the FMDV leader protease on LGP2. (A) HEK293 cells were mock-transfected (-) or co-transfected with a plasmid encoding *h*LGP2-Myc-DDK and empty vector (EV), LbWT or LbC51A. Cells were lysed 24 h later and analyzed by western blot for the indicated proteins with the specified antibodies. The N- and C-terminal cleavage products of LGP2 are indicated with arrows. (B) HEK293 cells were mock-transfected (-), transfected with EV, or with plasmids encoding LbWT or LbC51A and lysed 24 h later (left and middle panels); BHK-21 cells were mock-infected (-) or infected with FMDV O1BFS at an MOI of 5 and lysed 5 h later (right panel). Lysates were analyzed by western blot for detection of eIF4G cleavage products (arrowhead). (C) HEK293 cells were mock-transfected (-) or co-transfected with 500ng of a plasmid encoding DDK-METTL3 and 500ng of empty vector (EV), LbWT or LbC51A.

The FMDV-encoded Leader protease is an important virulence factor involved in IFN antagonism. Given that LGP2 is an innate immunity effector with synergistic effect on MDA5-induced antiviral response, we sought to determine whether FMDV is targeting LGP2 by a mechanism involving the activity of Lpro. First, we studied the effect of the co-expression on HEK293 cells of (Myc-DDK-tagged)-human LGP2 together with either the wildtype catalytically active form of Lbpro (LbWT) or LbC51A, an inactive form of the protease carrying a mutation in the active site [31,32]. The levels and integrity of LGP2 were analyzed 24 h later by immunoblot using antibodies against the N- or C-terminal regions of human LGP2, and compared to those observed after co-transfecting with the empty vector (EV) (Fig 1A). Expression of LbWT induced a drastic decrease in the full-length LGP2 levels. Interestingly, two LGP2-derived products of approximately 49 KDa and 27 KDa were specifically detected with the antibodies against the N- and C-terminal regions of LGP2, respectively. In contrast, when LGP2 and LbC51A were expressed together, no decrease in the helicase levels or additional bands were observed, suggesting that the LGP2 fragments detected may result from specific proteolytic cleavage by Lbpro.

Cleavage of eIF4G, a known cellular target protein for Lbpro was analyzed in HEK293 cells expressing the protease as a control of its catalytic activity in the experimental conditions used (Fig 1B). The 110 KDa cleavage fragment of eIF4G [33] was readily detected in cells expressing LbWT, like in cells infected with FMDV, in contrast to those expressing LbC51A (Fig 1B). The impact on the cap-dependent expression of a control DDK-tagged protein during co-expression with Lbpro due to eIF4G cleavage is shown for comparison (Fig 1C). In sum, these results suggest that LGP2 is a target for the proteolytic activity of the Leader protease.

**Fig 2:**
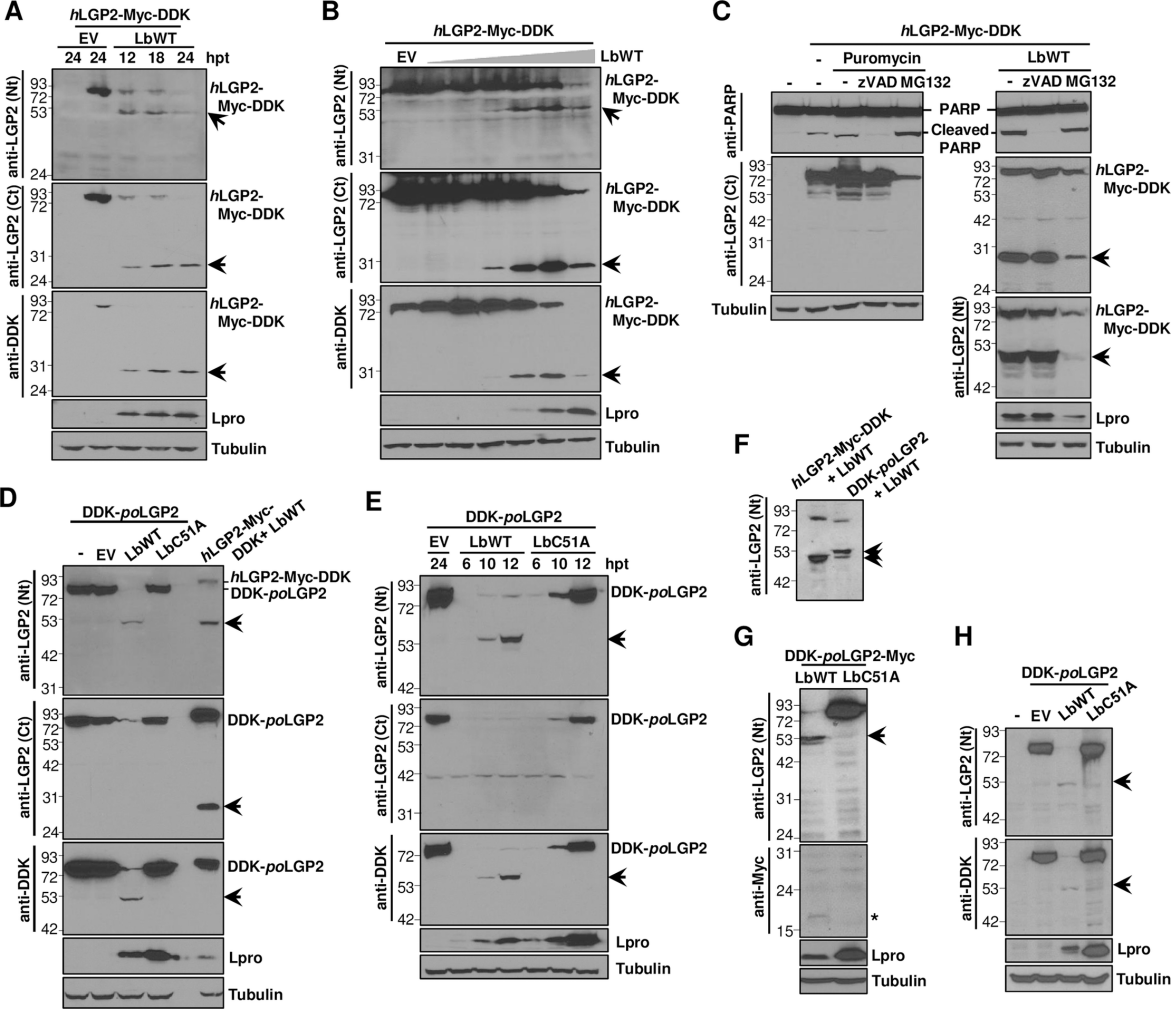
Expression of catalytically active FMDV Lbpro induces LGP2 cleavage. HEK293 cells were co-transfected with a plasmid encoding *h*LGP2-Myc-DDK and (A) LbWT or EV and lysed at the indicated times after transfection, (B) EV or increasing amounts of a plasmid encoding LbWT (0, 0.2, 2, 20, 200 or 2000 ng) and lysed 24 h later. (C) HEK293 cells were co-transfected with plasmids encoding *h*LGP2-Myc-DDK and LbWT in the presence of zVAD or MG132 caspase and proteasome inhibitors, respectively. In control cells, apoptosis was induced with puromycin. Cells were lysed 24 h later. (D) HEK293 cells were co-transfected with plasmids encoding DDK-*po*LGP2 and LbWT, LbC51A, EV or water (-). Control cells were co-transfected with plasmids encoding *h*LGP2-Myc-DDK and LbWT. (E) HEK293 cells were co-transfected with plasmids encoding DDK-*po*LGP2 and LbWT, LbC51A or EV and lysed at the indicated times after transfection. (F) HEK293 cells were co-transfected with plasmids encoding *h*LGP2-Myc-DDK or DDK-*po*LGP2 and LbWT and lysed 24 h later. (G) HEK293 cells were co-transfected with plasmids encoding DDK-*po*LGP2-Myc and LbWT or LbC51A and lysed 24 h later. (H) Porcine SK6 cells were mock-transfected (-) or co-transfected with plasmids encoding DDK-*po*LGP2 and LbWT, LbC51A or EV and lysed 24 h later. Cell lysates were analyzed by western blot for the indicated proteins using the specified antibodies. The N- and C-terminal cleavage products of LGP2 are indicated with arrows. A putative C-terminal cleavage product of *po*LGP2 harboring the Myc tag is indicated by an asterisk.

Next, we further characterized the Lbpro-LGP2 interaction. When lysates from cells co-expressing LGP2 and Lbpro were analyzed at different times after transfection, a progressive degradation of full-length LGP2, concomitant with detection of the N- and C-terminal products, could be observed (Fig 2A). The 27 KDa C-terminal fragment was also clearly detected with the anti-DDK antibody, consistent with the C-terminal location of the DDK tag in the LGP2 fusion protein transiently expressed. A dose-dependent effect of Lbpro on LGP2 was also observed, with accumulation of the N- and C-terminal products as higher concentrations of the protease were co-expressed with a fixed amount of the helicase (Fig 2B). With the highest amount of Lbpro assayed, the level of detection of all LGP2-derived bands 24 h after transfection decreased drastically, likely due to the extensive processing of the protein by Lb and subsequent degradation of the resulting products. The integrity of eIF4G in the lysates corresponding to the time course and Lbpro dose experiments was also analyzed to monitor the activityof Lbpro in each case (S1A and S1B Fig).

To address whether the caspase or the proteasome pathways were involved in LGP2 cleavage, the caspase inhibitor zVAD-FMK or the proteasome inhibitor MG132 was added to the transfection medium during LGP2 expression assays. As shown in Fig 2C, induction of apoptosis or proteasome did not result in LGP2 cleavage. In contrast, eIF4G analysis revealed the presence of the expected caspase-dependent fragments [34] (S1C Fig). Additionally, the 49 KDa N-terminal and 27 KDa C-terminal fragments were generated when LGP2 and Lbpro were co-expressed in the presence of the inhibitors. These results suggest that LGP2 cleavage was specifically attributable to the protease activity of the Lb protein and not a result of activation of cellular apoptosis and proteasome.

Given that pigs are among the most relevant natural host species for FMDV, we next assessed the ability of Lbpro to process porcine LGP2. For this purpose, we expressed the porcine helicase fused to an N-terminal DDK tag, as we failed in our attempts to detect the endogenous LGP2. Amino acid sequence alignment showed an 82% identity between human and porcine LGP2 proteins. Similarly to human LGP2, we found evidence of porcine LGP2 cleavage when it was co-expressed with the catalytically active form of Lbpro (Fig 2D and 2E). At 24 h after transfection, the full length porcine LGP2 was hardly detectable and no effect could be observed co-expressing the inactive form of the protease LbC51A (Fig 2D). Consistently, an N-terminal fragment of similar size to that generated from the human protein was readily detected with the antibody against the N-terminal region of LGP2 or the anti-tag antibody. The N-terminal LGP2 cleavage product generated by the activity of Lbpro accumulated over time after transfection (Fig 2E). The N-terminal fragment of porcine LGP2 showed a slightly slower migration than that derived from the human helicase (50 KDa approximately), consistent with the presence of the N-terminal DDK tag (Fig 2F).

Interestingly, no C-terminal products were found when using the specific antibody against the LGP2 C-terminal region in lysates from cells co-expressing the porcine helicase and LbWT (Fig 2D and 2E). To further address that issue and rule out any interference with the cross-reactivity of the antibody, raised against the C-terminal region of the human LGP2, we made a new construct for the expression of porcine LGP2 fused to a C-terminal Myc tag. Using an anti-Myc antibody we were unable to detect any LGP2-derived product around 27 KDa when porcine LGP2 and Lbpro were co-expressed, unlike that generated from human LGP2, and only a very faint product of approximately 18 KDa could be detected (Fig 2G). The cleavage pattern of porcine LGP2 was further confirmed in porcine SK6 cells (Fig 2H). The difference in the LGP2 C-terminal patterns observed between the human and porcine helicases induced by Lbpro might be the result of a more efficient degradation of the cleavage product generated from the porcine protein. Indeed, smaller degradation products can also be observed as the N-terminal fragments from both human and porcine LGP2 accumulate in transfected cells (Fig 2C, 2F and 2G). Additionally, secondary cleavage sites for Lbpro might be present in the C-terminal region of porcine LGP2.

Taken together, these results show that the FMDV Lbpro specifically cleaves human as well as porcine LGP2 when both helicase and protease are co-expressed in either human HEK293 or porcine SK6 cells.

### FMDV Lbpro interacts with LGP2

**Fig 3:**
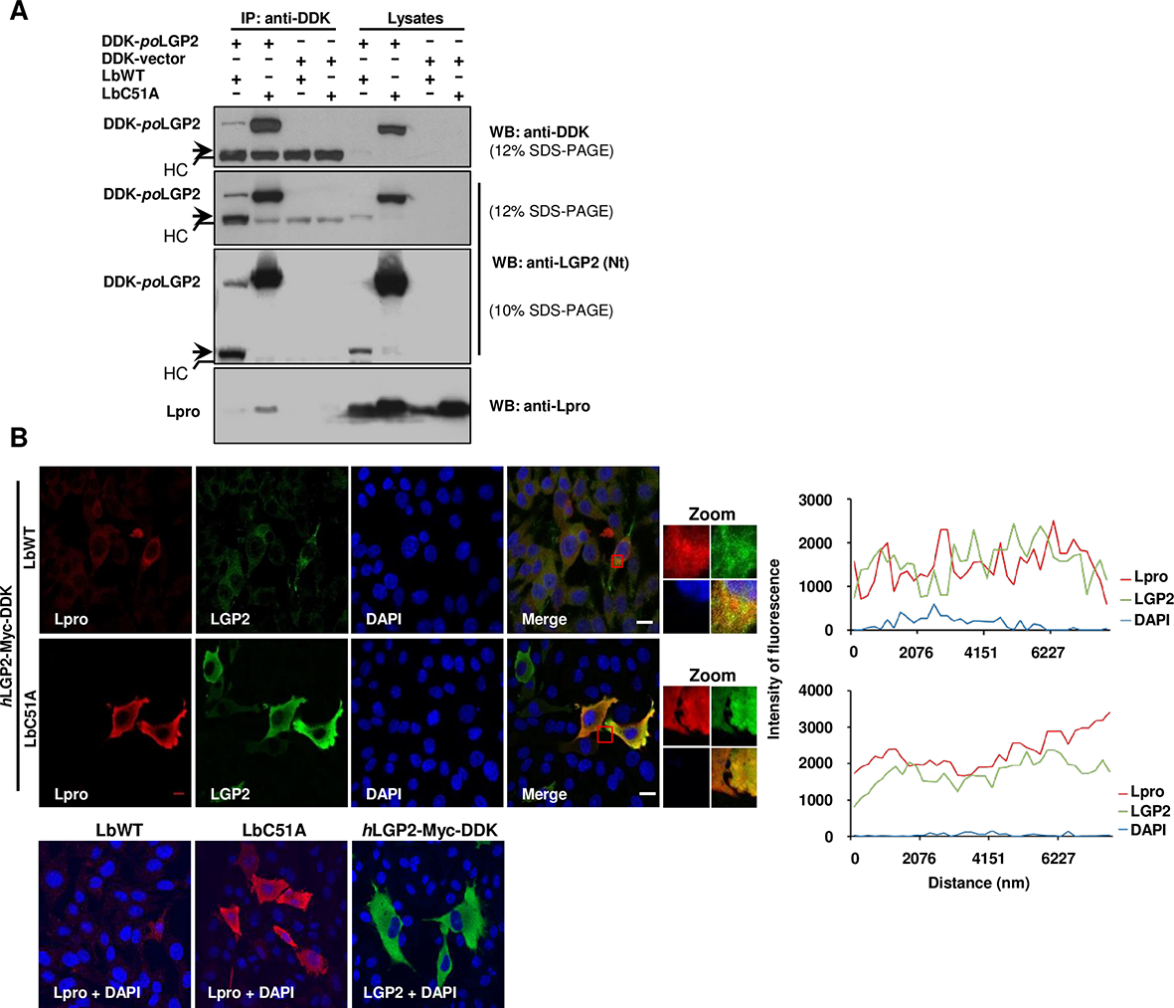
Interaction and cellular co-localization of Lbpro and LGP2. (A) SK6 cells were co-transfected with the indicated plasmids and lysed 24 h later. Lysates were subjected to IP and analyzed by western blot. The N-terminal cleavage product of LGP2 is indicated with an arrow. HC denotes the 50 KDa IgG heavy chain band. (B) Confocal microscopy images of BHK-21 cells at 20 h after co-transfection with plasmids encoding *h*LGP2-Myc-DDK and FMDV LbWT or LbC51A mutant. Control cells transfected with individual plasmids are shown (bottom). Primary antibodies used for LGP2 and Lb detection were a monoclonal anti-FLAG and a polyclonal anti-Lpro, respectively. Co-localization of LGP2 (green) and Lb (red) was assessed by histogram profiles of merged images. Nuclei were stained with DAPI. Scale bars, 10 μm.

Having established that LGP2 is a target for the catalytic activity of Lbpro, we sought to determine whether Lbpro interacts with LGP2. First, we carried out co-immunoprecipitation (coIP) assays in porcine SK6 cells co-expressing both proteins (Fig 3A). As expected, both full-length LGP2 and its N-terminal fragment were efficiently pulled down by the anti-tag antibody. Two different concentrations were used for SDS-PAGE analysis in order to separate the approximately 49 KDa LGP2 fragment from the 50 KDa IgG heavy chain band. We found that both LbWT and the inactive LbC51A mutant co-immunoprecipitated with LGP2, while no Lb was detected when co-expressed with the control tagged vector. According to the intensity of the LbWT and LbC51A bands in the corresponding IP fractions, it seems that both Lb forms are able to bind to full-length LGP2. As the amount of intact LGP2 24 h after co-transfection with LbWT is scarce, the low amount of protease immunoprecipitated with LGP2 detected as a faint band (Fig 3A). In contrast, LbC51A is accumulated in transfected cells and its interaction with LGP2 was readily detected. Additionally, the interaction between Lb and LGP2 might be abolished after cleavage contributing to a better detection of the LbC51A-LGP2 interaction. We also found by confocal microscopy that both LbWT as well as LbC51A co-localized with LGP2 when transiently co-expressed in BHK21 cells (Fig 3B). Taken together, these results suggest that the FMDV Lpro and LGP2 physically interact in vivo.

### LGP2 is cleaved during FMDV infection

**Fig 4:**
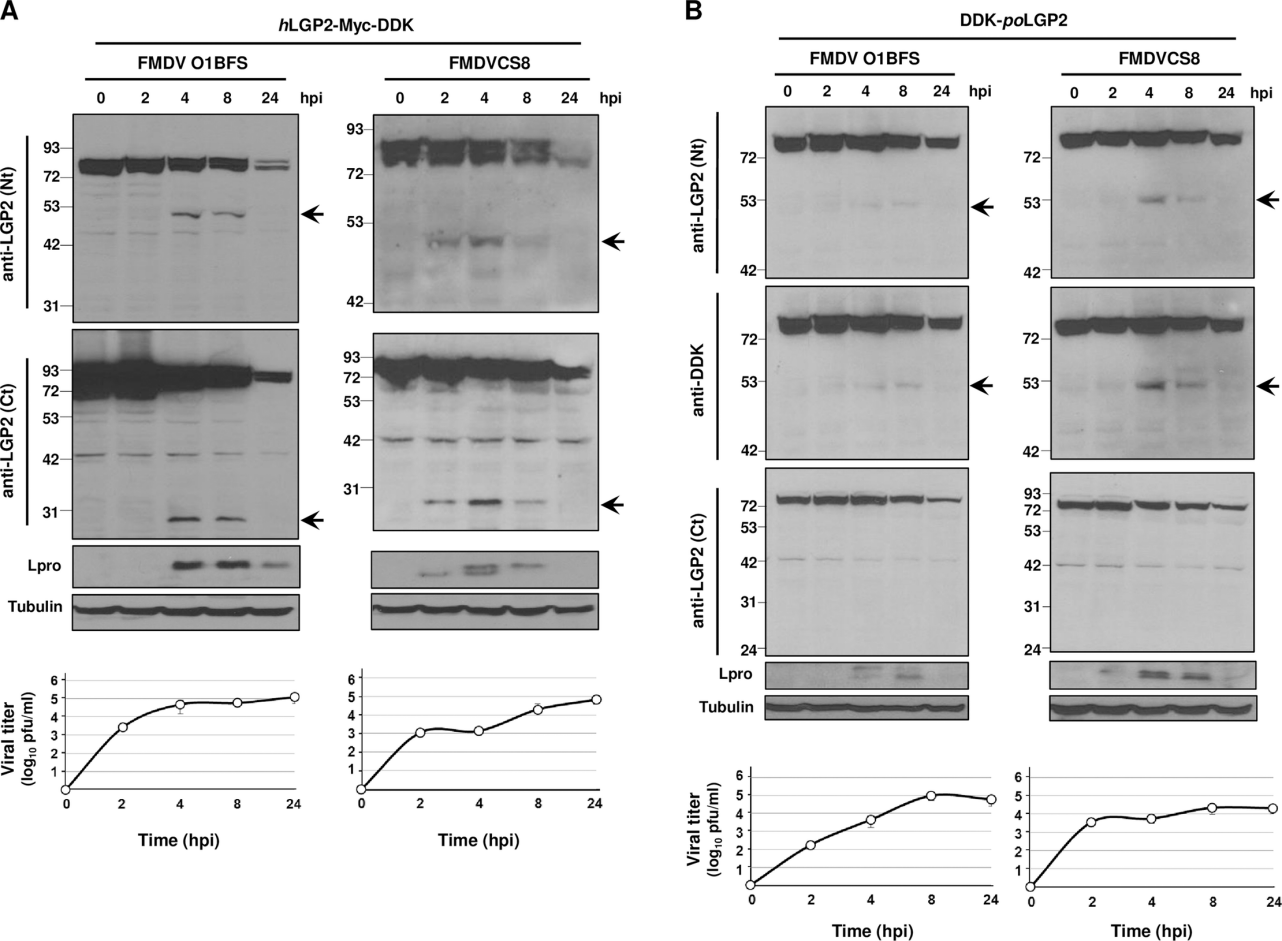
FMDV infection induces LGP2 cleavage. SK6 cells were transfected with a plasmid encoding (A) *h*LGP2-Myc-DDK or (B) DDK-*po*LGP2 and 24 h later infected with type-O or type-C FMDV isolates at an MOI of 5. Cells were lysed at different times after infection and analyzed by western blot using the indicated antibodies. Viral titers in the supernatants of transfected/infected cells at each time point are depicted. The N- and C-terminal cleavage products of LGP2 are indicated with arrows.

Having shown that Lbpro interacts with and cleaves LGP2, we hypothesized that the helicase cleavage event could play a role on viral host immune evasion. First, we determined whether FMDV infection induced LGP2 cleavage. For that purpose, human or porcine LGP2 was expressed in swine SK6 cells that were then infected with FMDV and lysed at different times after infection. Viruses in the supernatants collected from transfected and infected cells were titrated at the corresponding time points (Fig 4A and 4B). In these assays, two serologically and genetically divergent FMDV isolates were used: type-O O1BFS and type-C CS8. When SK6 cells expressing human LGP2 were infected with FMDV, the N- and C-terminal LGP2 cleavage products were clearly detected at 2 or 4 h post-infection (hpi) for CS8 or O1BFS isolates, respectively and up to 8 hpi (Fig 4A). When porcine LGP2 was expressed, FMDV infection with O1BFS or CS8 isolates generated the LGP2 N-terminal product of about 50 KDa which could be detected between 4–8 hpi using antibodies against either the N-terminal region of LGP2 or the DDK-tag (Fig 4B). Similarly to assays using ectopically expressed Lbpro, no C-terminal products derived from porcine LGP2 could be detected in human or swine cells. In all cases, detection of the human or porcine LGP2 cleavage products correlated with accumulation of the FMDV-encoded Lpro which could be often detected as a doublet, including Lb and a slower migrating form corresponding to Lab (Fig 4A and 4B). Also, LGP2 cleavage products detection coincided with times of higher viral titers (Fig 4A and 4B). These results show that LGP2 cleavage occurs during FMDV infection and the cleavage patterns observed are equivalent to those found in the above experiments using transiently expressed Lbpro (Fig 2). The impact of FMDV infection on eIF4G as result of Lpro activity on a known cellular target was monitored over time in SK6 cells transfected with human or porcine LGP2 (S2A and S2B Fig, respectively). While eIF4G cleavage was complete at 8 hpi (S2 Fig), full-length LGP2 was still abundant in cells (Fig 4A and 4B), likely due to a lower affinity for LGP2 together with the excess of overexpressed protein within cells at the time of infection.

Next, the pattern of LGP2 was analyzed at different times after infection with another aphthovirus, equine rhinitis A virus (ERAV). A drastic decrease in full-length LGP2 was observed after 24 h of infection and we were able to detect the 27 KDa C-terminal cleavage product at 48 hpi (S3 Fig). In contrast, during infection with different swine viruses causing a clinical disease similar to FMDV, like swine vesicular disease virus (SVDV)—a picornavirus -, or vesicular stomatitis virus (VSV)—a member of the *Rhabdoviridae* family, no cleavage products were detected and the levels of full-length LGP2 were maintained with no obvious decrease associated with infection (S4A and S4B Fig). Similar results were found when infection by two other distantly related picornaviruses—Aichivirus (Aiv) or encephalomyocarditis virus (EMCV)—was analyzed for LGP2 cleavage (S4C and S4D Fig). Again, no decrease in full-length LGP2 levels or any cleavage products could be detected. Interestingly, with the exception of ERAV, none of the picornaviruses analyzed express an active Leader protease. Altogether, these results suggest that LGP2 cleavage is not a general event during the course of infection by picornaviruses or vesicular swine viruses, but a specific mechanism occurring during FMDV infection and likely shared among aphthoviruses.

### Lbpro subverts the type-I IFN antiviral response promoted by LGP2

**Fig 5:**
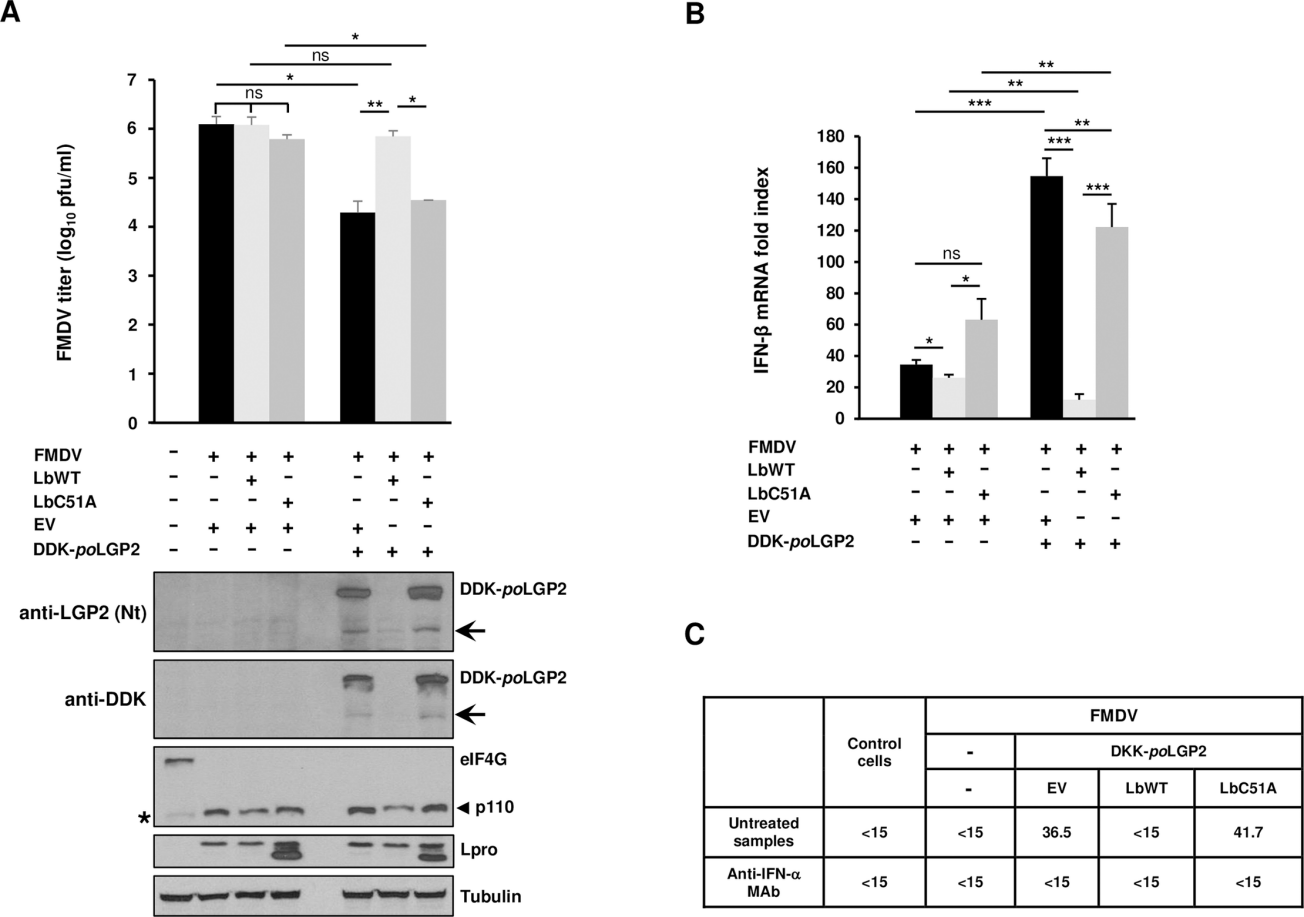
Effect of Lbpro and LGP2 co-expression on type-I IFN responses against FMDV infection. SK6 cells were co-transfected with a plasmid encoding DDK-*po*LGP2 or EV (2 μg) together with plasmids encoding LbWT, LbC51A or EV (1 μg). After 24 h, cells were infected with FMDV CS8 isolate at an MOI of 5. Supernatants were collected and cells lysed 8 h after infection. (A) Viral titers in supernatants were determined on IBRS2 cells. Data as mean ± SD of triplicates (n = 3). Cell lysates were analyzed by western blot for the indicated proteins. The N-terminal cleavage product of LGP2 is indicated with arrows. Bands corresponding to full-length eIF4G and the 110 KDa C-terminal cleavage fragment generated by Lpro are indicated. A minor band of slightly faster migration than p110 is observed in SK6 cells lysates and marked with an asterisk. (B) The fold induction of porcine IFN-β mRNA in cell lysates was determined by RT-qPCR normalized to GAPDH. Data as mean ± SD (n = 4). (C) IFN bioassay of supernatants. Antiviral activity is expressed as the reciprocal of the highest dilution needed to reduce the number of VSV plaques on IBRS-2 cells by 50%. When indicated, supernatants were previously treated with a monoclonal antibody anti-IFN-α. A representative IFN bioassay is shown. Student’s t test; *p < 0.05; **p < 0.01; ***p < 0.001; ns, not significant.

It has been shown that LGP2 overexpression negatively affects FMDV replication in cultured cells [30]. To determine whether LGP2 cleavage by Lpro is involved in IFN antagonism operating during FMDV infection, the effect of LGP2 and Lpro co-expression on the resulting viral titers, IFN-β mRNA levels and antiviral activity induced was analyzed in swine SK6 cells (Fig 5). First, the viral titers after 8 h of infection in cells co-expressing porcine LGP2 and either LbWT or inactive mutant LbC51A were compared (Fig 5A). As expected, expression of LGP2 induced a significant reduction in viral titers. Interestingly, co-expression of LbWT restored the viral titers recovered from control cells and no significant differences were found between cells co-expressing LGP2 and LbWT and those transfected with the EV alone or with LbWT or LbC51A independently. However, co-expression of LGP2 and LbC51A failed to have a stimulatory effect on FMDV replication and viral titers were equivalent to those obtained when LGP2 alone was expressed. When the integrity of LGP2 was analyzed, the N-terminal cleavage fragment could be detected in cells co-transfected with EV or LbC51A (Fig 5A), consistent with the cleavage pattern observed during infection (Fig 4). In contrast, no full-length LGP2 could be detected in cells co-expressing LbWT, suggesting an additive cleavage effect of overexpressed LbWT and FMDV-encoded Lpro on LGP2, though a putative contribution of the 3Cpro activity has not been analyzed and cannot be ruled out. In all cases, complete cleavage of eIF4G was observed, as expected at 8 h after infection (Fig 5A).

Since the Lpro activity is able to subvert the inhibitory effect mediated by LGP2 expression, ultimately promoting viral growth, we sought to address whether the mechanism behind that effect was related to type-I IFN induction. For that, the mRNA levels of porcine IFN-β were analyzed by RT-qPCR in SK6 cells transfected and infected as above (Fig 5B). Consistent with the different viral titers recovered from cells co-expressing porcine LGP2 and either LbWT or LbC51A, the IFN-β mRNA levels in cells co-transfected with LGP2 and LbWT were significantly lower than those measured in cells co-expressing LGP2 and either inactive LbC51A or an empty vector (100- and 128-fold lower, respectively) (Fig 5B). We also observed that lower levels of IFN-β mRNA were induced when LbC51A and LGP2 were co-expressed compared to cells co-expressing LGP2 and an EV. This could be due to interference by the inactive LbC51A, that is still able to bind LGP2, with the antiviral response triggered by the helicase. Additionally, a residual protease activity of the LbC51A, expressed at high levels in transfected cells, might contribute to the IFN reduction observed. In any case, this difference only seemed to induce a small insignificant reduction in viral titers.

Altogether, we concluded that, when LGP2 is transiently overexpressed, co-expression of catalytically active Lpro was associated with lower levels of IFN-β induction and higher levels of FMDV replication, in correlation also with complete cleavage of the helicase. Next, we aimed to determine whether the differences in IFN-β mRNA induction and FMDV titers described above correlated with different levels of antiviral activity present in the supernatants of the corresponding cells. Then, we carried out an IFN bioassay based on VSV infection inhibition in cells pre-treated with the supernatants from SK6 cells transfected and infected as above. The antiviral activity in each case reflects the protein levels of type-I IFN effectively secreted after mRNA induction and biologically active against viral infection. As shown in Fig 5C, antiviral activity was detected and measured in supernatants from cells co-transfected with LGP2 and either the EV or LbC51A but not with LbWT. This is consistent with the low levels of IFN-β mRNA measured by RT-qPCR and the higher FMDV titers recovered from those cells (Fig 5A and 5B). No antiviral activity could be detected in mock-transfected/infected or non-infected control cells either. The antiviral activity found in cells expressing LGP2 alone or together with inactive LbC51A was completely neutralized by incubation of the supernatants with a monoclonal antibody against porcine IFN-α, while no inhibitory effect on VSV infection was observed in untreated cells, proving the specificity of the neutralizations observed and further confirming that the antiviral activity subverted by Lbpro was indeed type-I IFN specific.

### Identification of the Lbpro cleavage site in LGP2

**Fig 6:**
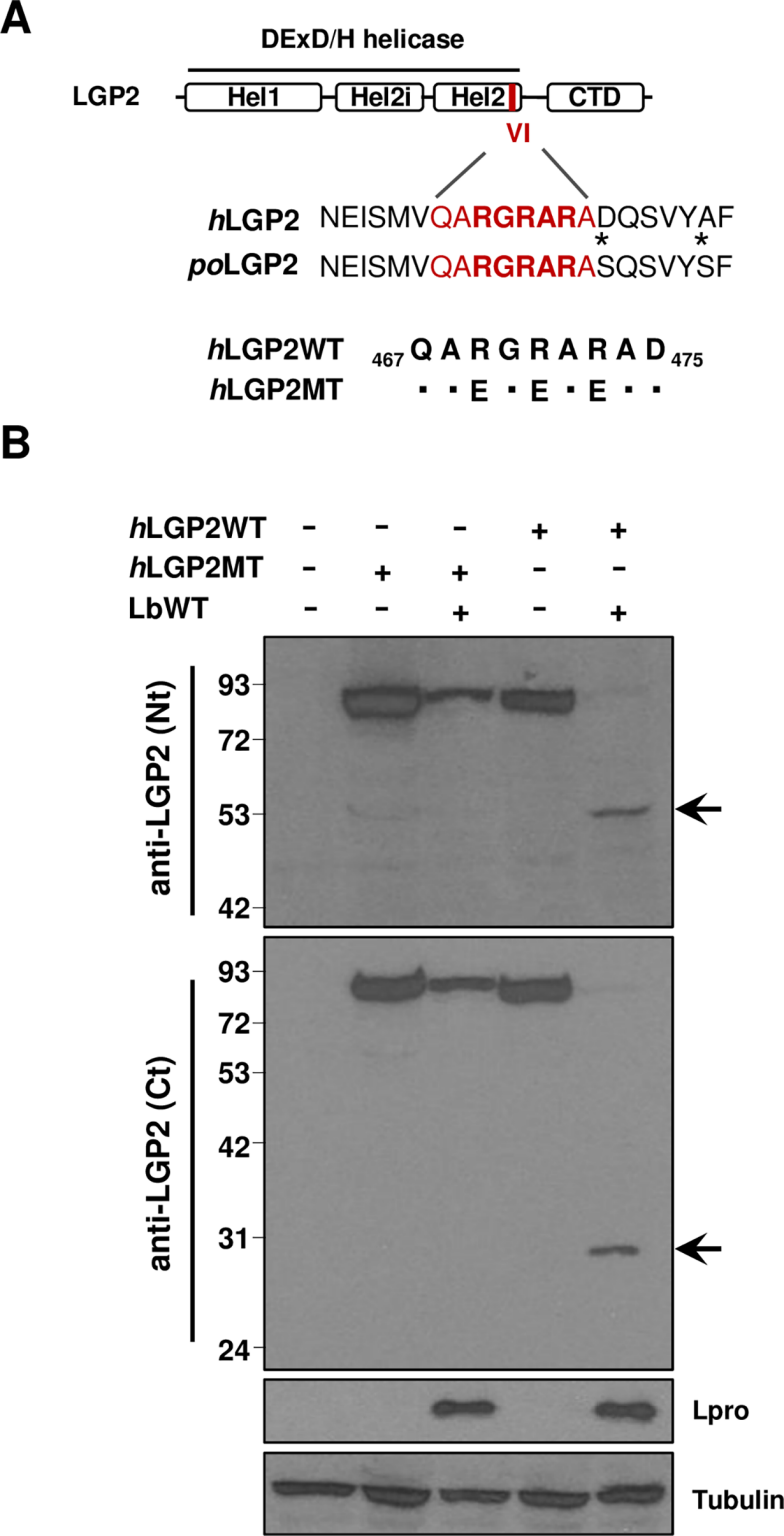
Mutation of the putative target sequence in LGP2 abolishes cleavage by Lbpro. (A) Schematic representation of LGP2 showing the three subdomains in the conserved DExD/H helicase domain (Hel1, Hel2i and Hel2) and the C-terminal domain (CTD). The position and sequence of the conserved helicase motif VI (in red) and surrounding residues in the human and porcine proteins are indicated. Amino acid substitutions in the mutant version of *h*LGP2 (*h*LGP2MT) compared with WT sequence are indicated in the corresponding positions. (B) HEK293 cells were transfected with a plasmid encoding *h*LGP2-Myc-DDK (*h*LGP2WT) or its mutant version as indicated above (*h*LGP2MT) either alone or together with a plasmid encoding LbWT. The amount of DNA in all transfections was balanced with EV (-). Cell lysates were analyzed 24 h later by western blot for the indicated proteins using the specified antibodies. The N- and C-terminal cleavage products of LGP2 are indicated with arrows.

Though several cellular proteins are known targets for Lpro cleavage, only a few substrate sequences which, however, do not share a unique amino acid sequence motif, have been identified experimentally. Given that the estimated molecular weights of the N- and C-terminal fragments generated after Lbpro cleavage of human LGP2 seem to add up to the size of the full-length protein (77 KDa), suggesting a single cleavage site, and the similar size observed for the N-terminal fragment cleaved from the porcine protein (about 50 KDa), the amino acid sequence around the putative cleavage region was analyzed for similarities with previously reported Lpro target sequences. A stretch of positively charged R amino acids (RGRAR) resembling the (R)(R/K)(L/A)(R) target motif defined for Gemin5 and Daxx (death-domain associated protein) proteins [35] was identified in the conserved helicase motif VI of both human and porcine LGP2 sequences (Fig 6A). To determine the relevance of the arginine residues in the candidate target sequence, a triple substitution mutant to negatively charged glutamic acid (RGRAR/EGEAE) was generated in the human protein (Fig 6A). Unlike LGP2WT, when the LGP2 triple mutant (LGP2MT) was co-transfected with Lbpro no cleavage products could be detected, though the expected decrease in the cap-dependent expression of the tagged-polypeptide, concomitant with Lpro expression was evident (Fig 6B). These results suggest that residues 69–73 in human LGP2 (corresponding to amino acids 72–76 in the porcine protein) are a target motif for Lpro proteolytic activity.

## Discussion

In this study, we identified the innate immune sensor LGP2 as a target for the FMDV Leader protease. The IFN system is a powerful component of the antiviral response and viruses have evolved sophisticated strategies to evade the host innate immune response by interfering with the different events involved in PRR activation and signaling [15,16]. FMDV is no exception, and viral proteases have been found to counteract the innate responses induced in cells during the course of infection [17,18]. Lpro is known to prevent the host antiviral response by several mechanisms including cleavage of initiation factor eIF4G - and then prevention of the synthesis of IFN and other cytokines immediately after infection-, degradation of NF-κB, and deubiquitination of immune signaling molecules [18,24]. Though the contribution of LGP2 to innate immune activation is still not fully understood, recent work unveils a relevant regulatory role on RLR signaling through CARD-independent interactions. The dsRNA generated during picornavirus replication is sensed by MDA5, and LGP2 is believed to promote the viral RNA-MDA5 interaction leading to efficient antiviral signaling [36]. Indeed, RNA derived from EMCV infection with strong MDA5-stimulatory activity was immunoprecipitated with LGP2 [37]. It is also known that either LGP2 or MDA5 deficiency results in higher susceptibility to picornavirus infections [38,39]. A recent report shows that, in the absence of infection or viral proteins, LGP2 functioned as a biphasic master activator of numerous innate immunity genes, sequentially induced in a cascade fashion leading to production of IFN. In turn, LGP2 was subject to negative control by cellular translation regulators [40].

Here, we first provide evidence that LGP2 is cleaved by the FMDV Lbpro resulting in a drastic decrease of full-length LGP2 and accumulation of specific cleavage products in an Lb-dose-dependent manner. Catalytically inactive mutant LbC51A had no effect on LGP2. We found that LGP2 cleavage was specifically attributable to Lbpro catalytic activity and not a result of activation of cellular apoptosis or proteasome. When the patterns of human and porcine LGP2 after co-expression with Lbpro were compared, a similar N-terminal fragment of about 50 KDa was observed in both cases, while an additional C-terminal fragment of 27 KDa was only detected for human LGP2.

Though several cellular proteins are known targets for Lpro cleavage, only a few substrate sequences have been identified experimentally and there is no consensus for Lpro target sequence. Together with the L/VP4 junction of the viral polyprotein, cleavage sites in eIF4GI, eIF4GII, Gemin5 and Daxx have been determined. The analysis of the amino acid sequence around the putative cleavage region revealed a conserved motif in human and porcine LGP2 proteins resembling the Lpro target site (R)(R/K)(L/A)(R) reported in Gemin5 and Daxx [35]. Replacement of all three positively charged R residues by E (RGAR/EGEAE) in human LGP2 protein completely abolished cleavage by Lbpro, defining the RGRAR sequence in the conserved helicase motif VI as the FMDV Leader protease target site in LGP2. This result further increases the number of host factors cleaved by this protease, opening new avenues for the identification of novel targets. Cleavage at that position would excise a 27 KDa C-terminal fragment, in agreement with our results, involving removal of part of the helicase domain and the complete CTD region. An intact CTD is required for RNA specific recognition, and LGP2 mutants in the CTD, helicase domain or both are known to be RNA binding-deficient and have poor enhancing activity towards MDA5 [7]. Thus, cleavage by Lpro would likely abolish the antiviral function of LGP2.

Additionally, we found that both active LbWT and inactive LbC51A were able to interact with LGP2, though LbC51A co-immunoprecipitated with the helicase more efficiently than LbWT. Besides the higher levels of expression achieved with the inactive version of the protease, these results suggest that Lbpro forms a transient interaction with LGP2 that is abolished after LGP2 cleavage. This is also consistent with the low levels of full-length LGP2 and high levels of the N-terminal fragment present in the lysates when LbWT was expressed.

Given that LGP2 cleavage by Lpro was found using the ectopically expressed protease, we analyzed the fate of overexpressed LGP2 during FMDV infection in order to address the biological relevance of the cleavage event. Interestingly, the human and porcine helicase patterns observed during FMDV infection with two different viral isolates overlapped with those observed under LbWT overexpression. Consistently, the corresponding N- and C-terminal LGP2 cleavage products accumulated in cells around 4 h after infection, coinciding with accumulation of viral Lpro, and thus supporting that LGP2 is processed by Lpro during FMDV infection.

Evidence of LGP2 cleavage was also found during infection with the equine aphthovirus ERAV, which shares with FMDV, unlike most picornaviruses, the presence of a proteolytically active Leader protein at the N-terminus of the polyprotein [41]. In contrast, no evidence of LGP2 cleavage or noticeable decrease was observed during infection with a set of different viruses, including unrelated picornaviruses AiV and EMCV, and other RNA viruses causing swine vesicular disease similar to FMD like SVDV—picornavirus—and VSV—rhabdovirus, suggesting that no evident specific mechanisms targeting LGP2 integrity were exerted by these other viruses. In agreement with these data, Feng et al. could not find any sign of LGP2 cleavage in HeLa cells during infection with Coxsackievirus B3, a subtype of Enterovirus B, another picornavirus [42]. Interestingly, Gemin5 cleavage by Lpro was only observed in FMDV-infected cells but not during infection with picornaviruses belonging to different genera like SVDV or EMCV [35].

A previous study showed a detrimental effect of LGP2 expression on FMDV replication, as well as a decrease in the helicase levels when different viral proteins—including Lpro, 3C and 2B - were expressed [30]. The authors concluded that 2B interaction with LGP2 was responsible for this effect and linked it to regulation of the inflammatory response in infected cells by an unknown mechanism. In this study, we showed that LGP2 cleavage by Lpro is involved in IFN antagonism operating during FMDV infection. Expression of catalytically active Lpro was able to subvert the IFN-β induction and inhibitory effect on viral growth mediated by LGP2, circumventing the type-I IFN specific antiviral activity induced in porcine cells that had been transfected and later infected with FMDV.

A potential role on LGP2 antagonism has been suggested for the binding of paramyxovirus V and HCV NS3proteins to the helicase domain of LGP2. LGP2-V protein interaction disrupts the ATP hydrolysis activity of LGP2 [28]. The HCV NS3 protein has a protease domain at its N-terminus linked to a C-terminal helicase domain. LGP2-NS3 interaction might contribute to localize NS3 to mitochondria for MAVS cleavage [29]. To our knowledge, this is the first report of an LGP2 cleavage event involving a virally encoded protein, with implications in the resulting type-I IFN response of the host against viral infection. FMDV is highly sensitive to IFN, and IFN-based strategies have proved to be efficient biotherapeutic approaches against the virus [43–45]. Cleavage of LGP2 by the Leader protease unveils a new antagonistic mechanism evolved by FMDV, and likely other aphthoviruses, directed to suppress the host IFN response. LGP2 cleavage expands the list of cellular proteins involved in immune response targeted by the FMDV Lpro, highlighting its relevance as a crucial proteolytic virulence factor. The suppressor effect exerted by Lpro on the LGP2-dependent type-I IFN induction may be the result of blockade at several points of the pleiotropic activation of innate responses orchestrated by LGP2, included its synergistic effect on MDA5 signaling. Our findings encourage further studies focused on the role of LGP2 on the antiviral response against FMDV and the putative involvement of MDA5 in the LGP2-Lpro interplay. A better understanding of the mechanisms employed by viruses to circumvent the host antiviral signaling will contribute to development of new therapeutic strategies to fight viral infections, including antiviral approaches and novel vaccines. This is of particular relevance for FMDV, given the rapid spread of the virus and the devastating economic consequences associated with FMD outbreaks.

## Materials and methods

### Cells and viruses

HEK293, Vero and BHK21 cells (all three from ATTC) and SK6 and IBRS-2 cells (both obtained from Centro de Investigación en Sanidad Animal, CISA-INIA, Madrid, Spain) were all cultured in Dulbecco’s modified Eagle’s medium (DMEM; GIBCO) supplemented with 10% fetal bovine serum at 37 ^o^C with 5% CO_2_. These cell lines were used for propagation and infection assays of the corresponding viruses.

FMDV type-C CS8 and type-O O1BFS isolates were propagated in swine SK6 or IBRS-2 cells. ERAV and Aichivirus were propagated in Vero cells. SVDV was propagated in SK6 cells. VSV was propagated in IBRS-2 cells.

### Plasmids and transfection

Plasmids encoding the WT or C51A mutant Lb protease were generated by PCR amplification of the corresponding regions from an FMDV O1K full-length cDNA clone [46] and insertion into the *Bam*HI and *Xba*I sites of pcDNA3.1(+) (Invitrogen). Plasmids encoding the sequence of porcine LGP2 with a C-terminal Myc tag and/or an N-terminal DDK tag were generated by gene synthesis (NZYTech) and cloning into the *Nhe*I and *Xba*I sites of pcDNA3.1(+) (Invitrogen). Plasmid encoding (C-terminal Myc-DDK-tagged)-human LGP2 was from Origen. Plasmid pcDNA3/Flag-METTL3 encoding human methyltransferase-like 3 was from Addgene (# 53739).

For transfection, 2 μg of LGP2-encoding plasmids and 1 μg of plasmids encoding FMDV proteases were used using Lipofectamine 2000 (Invitrogen) following the manufacturer's recommendations. The total amount of transfected DNA was balanced to 3 μg with empty vector. In some experiments, the transfection medium was supplemented with 20 μM Puromycin (apoptosis inducer, Sigma-Aldrich), 20 μM zVAD-FMK (broad caspase inhibitor, Promega) or 10 μM MG132 (proteasome inhibitor, Cayman Chemical).

### Antibodies

Mouse monoclonal anti-LGP2 (E-1, raised against a peptide mapping at the C-terminus of human LGP2) and goat polyclonal anti-LGP2 (N-14, raised against a peptide mapping near the N-terminus of human LGP2) were purchased from Santa Cruz Biotechnology Inc. Mouse monoclonal anti-FLAG (M2) was purchased from Sigma-Aldrich. Rabbit polyclonal anti-PARP and mouse monoclonal anti-cleaved PARP (Asp214) (19F4) were purchased from Cell Signaling Technology. Mouse monoclonal anti-Pig IFN-Alpha (K9) was purchased from PBL Assay Science. Rabbit polyclonal anti-FMDV Leader protease was raised against the Lab/Lb fusion protein expressed by pE16 plasmid [47] and kindly provided by Ewald Beck. Rabbit polyclonal anti-βII-tubulin [48] was achieved from Sobrino F Lab. Mouse monoclonal anti-G3BP (clone 23) was purchased from BD Biosciences. Goat polyclonal anti-eIF4G (D-20) antibody was purchased from Santa Cruz Biotechnology Inc. Goat anti-mouse, goat anti-rabbit and rabbit anti-goat IgG (H + L) secondary antibodies HRP conjugate were purchased from Thermo Scientific.

### Quantification of viral growth

Cells were transfected with indicated plasmids with Lipofectamine 2000 (Invitrogen) according to the manufacture’s protocol and 24 h later, cells were infected with the corresponding viruses. At different times after infection, supernatants were harvested, serially diluted and viral titers were determined by plaque assay on fresh monolayers. After 1 h of infection, cells were washed twice and overlaid with 0.5% agar. After 24 h, cells were fixed with 10% formalin and stained with crystal violet. Viral titers were expressed as plaque forming unit (pfu)/ml. The mean values and standard deviations were calculated from triplicate determinations.

### SDS-PAGE and western blot

Cells were harvested after transfection or infection, and lysed with PBS containing 1% NP-40, 1 mM DTT and protease inhibitor cocktail (Complete, Roche). Whole cell lysates were incubated at room temperature for 5 min and cleared by centrifugation at 9.300 x *g* for 5 min at 4°C. Protein concentrations were determined based on the Bradford method using the Bio-Rad protein assay kit. Equal amounts of proteins (20–50 μg) were separated by 6–12% SDS-PAGE and electrophoretically transferred onto a nitrocellulose membrane (GE Healthcare). After blocking with 3% non-fat milk in 0.05% Tween20 PBS, the membranes were incubated with the primary antibodies, followed by horseradish peroxidase-conjugated goat anti-rabbit, anti-mouse or rabbit anti-goat IgG. Membrane bound antibodies were detected by enhanced chemiluminescent luminol substrate (Western Lightning Plus Chemiluminescent Substrate kit, Perkin Elmer) and visualized by exposure to X-ray films.

### In vivo CoIP

1x10^6^ SK6 cells were co-transfected with 2 μg of DDK-*po*LGP2 or DDK-vector plasmids together with 1 μg of plasmids encoding LbWT or LbC51A. Cells were harvested 24 h later and lysed with 100 μl of lysis buffer (50 mMTris-HCl [pH, 7.5], 150 mMNaCl, 0.5% NP-40, and protease inhibitor cocktail). The supernatants were collected by centrifugation at 10,000 x *g* for 5 min at 4°C and precleared with 25 μl of protein G-Agarose (Roche) for 1 h at 4°C with rotation. Proteins were immunoprecipitated by addition of monoclonal anti DDK and incubation for 4 h at 4°C and then, addition of protein A agarose and incubation at 4°C for 16 h. Immunoprecipitated complexes were washed three times with 400 μl of wash buffer 1 (0.1% NP-40, 50 mM Tris pH 7.5, 150 mM NaCl) and once with 400 μl of wash buffer 2 (50 mM Tris pH 7.5, 150 mM NaCl). Then, beads were collected via centrifugation at 10,000 x *g* for 2 min at 4°C, boiled at 100°C for 3 min in SDS protein-loading buffer and analyzed via WB.

### Confocal microscopy

BHK-21 cells were transfected with indicated plasmids with Lipofectamine 2000 (Invitrogen) according to the manufacture’s protocol. After 20 h, cells were washed three times with room temperature PBS, then fixed with 4% paraformaldehyde solution in PBS for 20 min, washed again with PBS three times and permeabilized with 0.05% Tween in PBS for 15 min. After wash three times with PBS, cells were blocked with 5% BSA in PBS for 1 h. Cells then were incubated with specific primary antibodies overnight at 4°C, followed by incubation for 1h with goat anti-mouse Alexa fluor 488 and goat anti-rabbit Alexa fluor 647 as secondary antibody. Nuclei were stained with 4',6-diamidino-2-phenylindole (DAPI) at 1 μg/ml. Images were acquired with a Zeiss LSM 880 Meta confocal microscope (Carl Zeiss Microimaging, Thornwood, NY) with a Plan Apochromatic ×40/1.4 oil objective lens. Image processing and analysis of intensity of fluorescence by histograms were carried out using Fiji/ImageJ software.

### Quantitative reverse transcription (RT)-PCR

Total RNA was isolated from SK6 cells using TriReagent (Sigma), quantified by spectrometry and DNase-treated with Turbo DNA-free kit (Ambion). 500 ng of RNA was used for RT with 20U of SuperScript III RT (Invitrogen) at 55 ^o^C for 30min. Quantitative PCR was carried out using aliquots of the RT reactions (1/10) and LightCycler FastStart DNA master SYBR green I (Roche). All reactions were conducted in triplicate. Data were analyzed using the ΔΔ*C*_*T*_ method. IFN-β gene expression was normalized to that of the GAPDH and was expressed as the fold increase above the level of mock-transfected cells. Primers for amplification of IFN-β and GAPDH have been previously described [49].

### Antiviral activity

The antiviral activity in supernatants from transfected and/or infected SK-6 cells was determined by a VSV infection inhibition assay on IBRS-2 cells (IFN bioassay) as described [49]. Briefly, after transfection, SK-6 cells were incubated at 37°C and 24 h later infected with FMDV CS8 isolate at an MOI of 5. Supernatants were collected 7 h later and infectious particles were inactivated by acidic treatment (pH = 2–3) with 10M HCl for 16–20 h at 4°C and then, neutralized (pH = 7) with 10 M NaOH. Dilutions of the treated supernatants (up to 1/15) were added on fresh IBRS2 monolayers and incubated for 24 h at 37°C. Then, cells were washed and infected with VSV (50–60 pfu per 1x10^6^ cells) and plaques were counted 24 h after infection. Antiviral activity was defined as the reciprocal of the highest dilution resulting in a 50% reduction in the number of plaques relative to untreated cells. In order to block the antiviral activity exerted by IFN-α in SK6 cells supernatants on VSV infection, some samples were incubated for 1 h at 37°C with 1μg of a monoclonal antibody against swine IFN-α (clone K9) from PBL Assay Science.

### Statistical analysis

The unpaired Student’s *t* test for independent samples was used to compare data using IBM SPSS Statistical (v.24) software; a p value of < 0.05 was considered statistically significant and a p value of > 0.05 was considered statistically non-significant. In all graphs, three asterisks indicate a p value of <0.001, two asterisks indicate a p value of <0.01, one asterisk indicates a p value of <0.05, and ns indicates not significant (p > 0.05). The number of replicates used in experiments is specified in the corresponding figure legends.

## Supporting information

S1 Fig Analysis of the integrity of eIF4G in HEK293 cells co-expressing *h*LGP2 and Lbpro. Panels A, B and C show the analysis by western blot with an anti-eIF4G antibody of the lysates shown in Fig 2A, 2B and 2C, respectively. Bands corresponding to full-length eIF4G and the 110 KDa C-terminal cleavage fragment generated by Lbpro are indicated. In panel C, caspase-specific cleavage products are marked with asterisks.
(PDF)

S2 Fig Analysis of the integrity of eIF4G in SK6 cells expressing *h*- or *po*-LGP2 and infected with FMDV. Panels A and B show the analysis by western blot with an anti-eIF4G antibody of the lysates shown in Fig 4A and 4B, respectively. Bands corresponding to full-length eIF4G and the 110 KDa C-terminal cleavage fragment generated by Lpro are indicated. A minor band of slightly faster migration than p110 is observed in SK6 cells lysates and marked with an asterisk.
(PDF)

S3 Fig LGP2 is cleaved during ERAV infection. Vero cells were transfected with a plasmid encoding *h*LGP2-Myc-DDK and 24 h later infected with ERAV at an MOI of 1. Cells were lysed at different times after infection and analyzed by western blot using the indicated antibodies. Viral titers in the supernatants of transfected/infected cells at each time point are depicted. The C-terminal cleavage product of LGP2 is indicated with an arrow.
(PDF)

S4 Fig LGP2 degradation is not observed during infection with other picornaviruses and swine vesicular viruses. SK6 cells were transfected with a plasmid encoding *h*LGP2-Myc-DDK or DDK-*po*LGP2 and 24 h later infected with (A) SVDV or (B) VSV at an MOI of 5. (C) Vero cells were transfected with a plasmid encoding *h*LGP2-Myc-DDK and 24 h later infected with Aichivirus at an MOI of 5. (D) BHK-21 cells were transfected with a plasmid encoding *h*LGP2-Myc-DDK and 24 h later infected with EMCV at an MOI of 5. Cells were lysed at different times after infection and analyzed by western blot using the indicated antibodies. Viral titers in the supernatants of transfected/infected cells at each time point are depicted.
(PDF)
