# Supplementary figures and images for: Innate immune sensor LGP2 is cleaved by the Leader protease of foot-and-mouth disease virus
Source: PLoS Pathog. 2018 Jun 29;14(6):e1007135. doi: 10.1371/journal.ppat.1007135 (PMC6042790; doi:10.1371/journal.ppat.1007135)

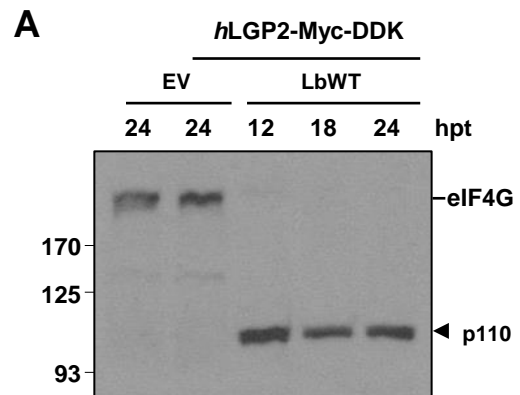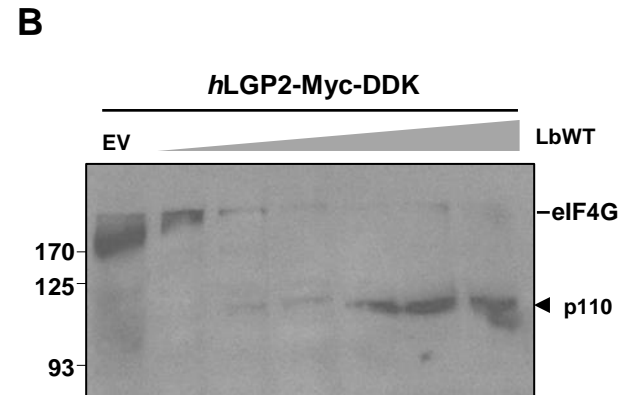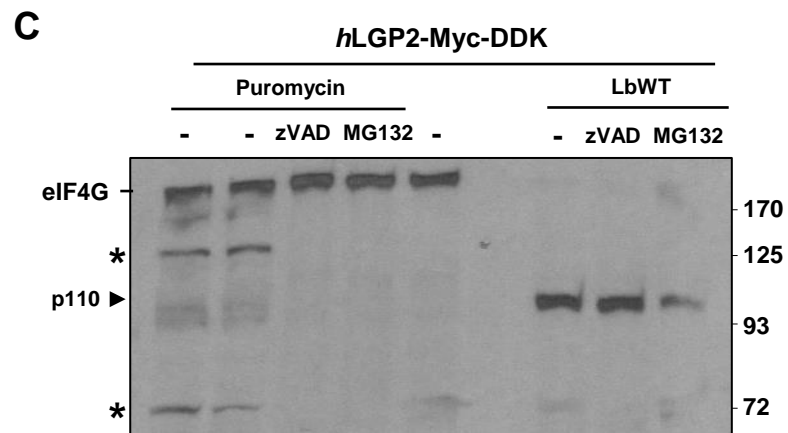

Supplement: S1 Fig — Panels A, B and C show the analysis by western blot with an anti-eIF4G antibody of the lysates shown in Fig 2A, 2B and 2C, respectively. Bands corresponding to full-length eIF4G and the 110 KDa C-terminal cleavage fragment generated by Lbpro are indicated. In panel C, caspase-specific cleavage products are marked with asterisks. (PDF) [file ppat.1007135.s001.pdf]

**A**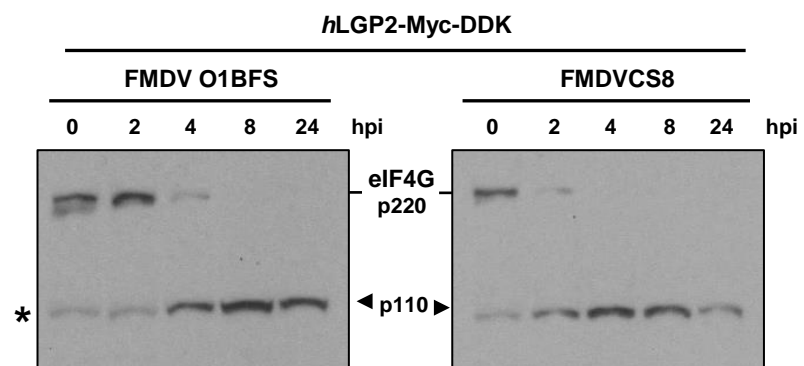**B**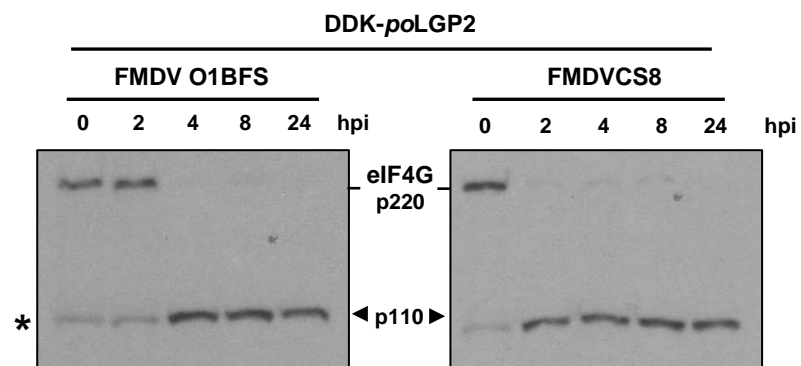

Supplement: S2 Fig — Panels A and B show the analysis by western blot with an anti-eIF4G antibody of the lysates shown in Fig 4A and 4B, respectively. Bands corresponding to full-length eIF4G and the 110 KDa C-terminal cleavage fragment generated by Lpro are indicated. A minor band of slightly faster migration than p110 is observed in SK6 cells lysates and marked with an asterisk. (PDF) [file ppat.1007135.s002.pdf]

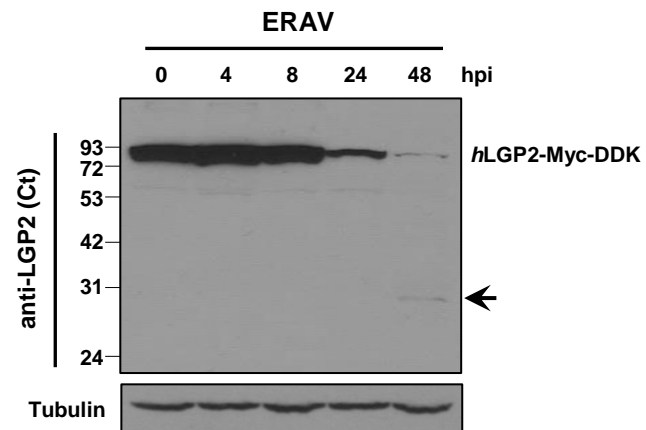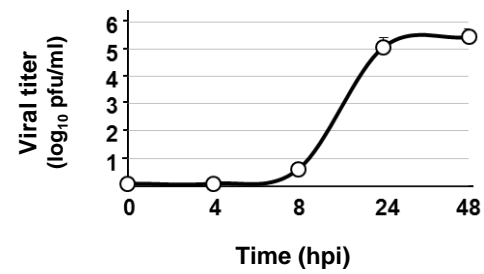

Supplement: S3 Fig — Vero cells were transfected with a plasmid encoding hLGP2-Myc-DDK and 24 h later infected with ERAV at an MOI of 1. Cells were lysed at different times after infection and analyzed by western blot using the indicated antibodies. Viral titers in the supernatants of transfected/infected cells at each time point are depicted. The C-terminal cleavage product of LGP2 is indicated with an arrow. (PDF) [file ppat.1007135.s003.pdf]

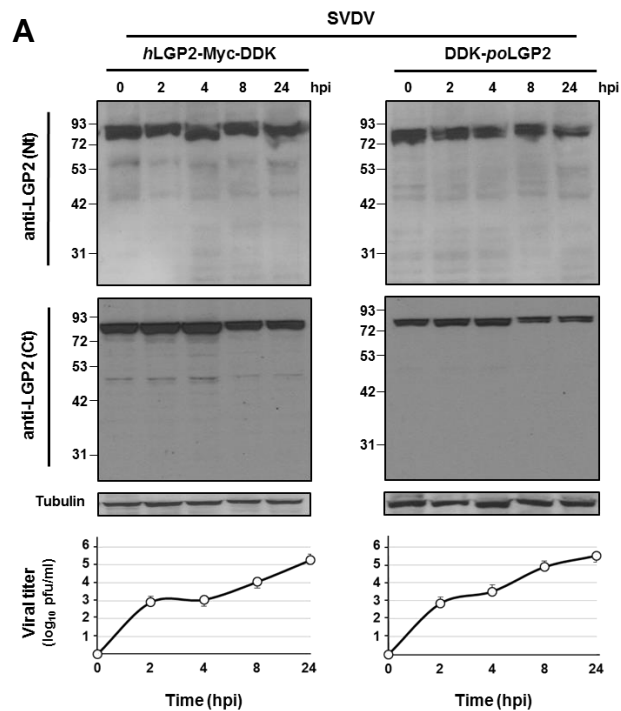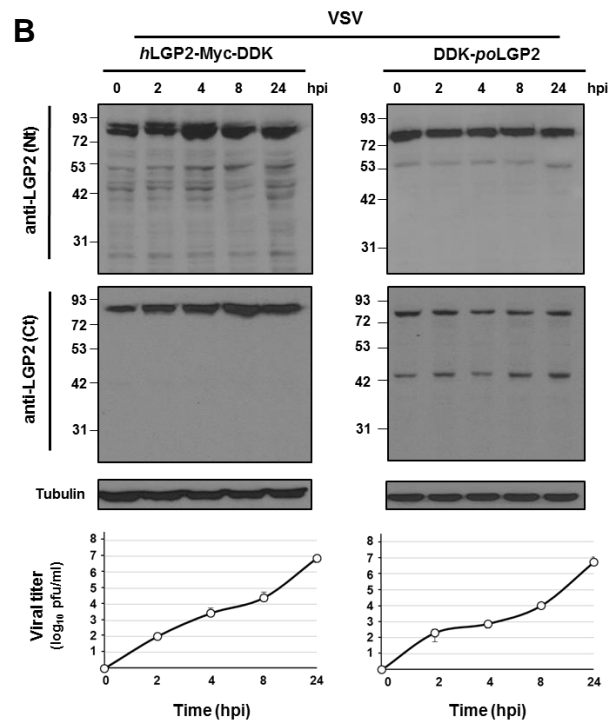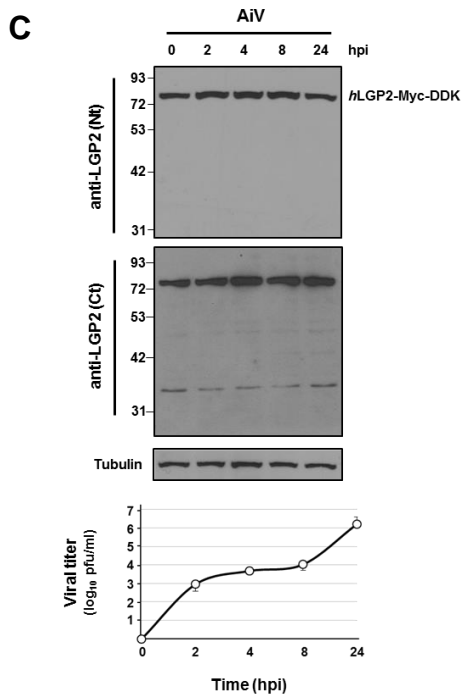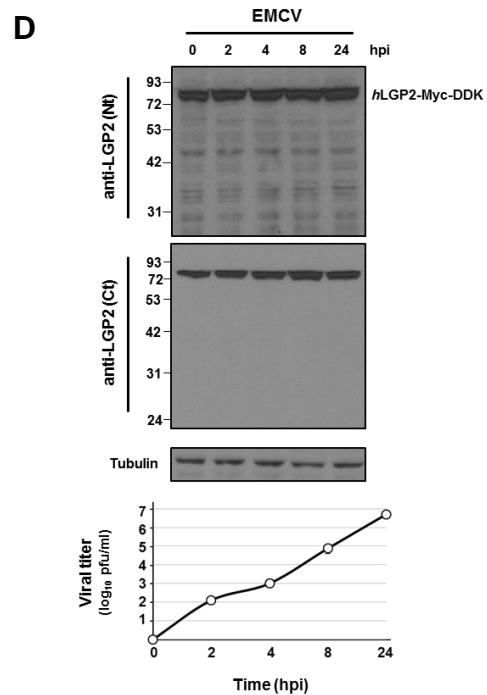

Supplement: S4 Fig — SK6 cells were transfected with a plasmid encoding hLGP2-Myc-DDK or DDK-poLGP2 and 24 h later infected with (A) SVDV or (B) VSV at an MOI of 5. (C) Vero cells were transfected with a plasmid encoding hLGP2-Myc-DDK and 24 h later infected with Aichivirus at an MOI of 5. (D) BHK-21 cells were transfected with a plasmid encoding hLGP2-Myc-DDK and 24 h later infected with EMCV at an MOI of 5. Cells were lysed at different times after infection and analyzed by western blot using the indicated antibodies. Viral titers in the supernatants of transfected/infected cells at each time point are depicted. (PDF) [file ppat.1007135.s004.pdf]
